# Measuring the Attitudes of Animal Hospital Staff Toward Animals in Türkiye

**DOI:** 10.3390/ani16060888

**Published:** 2026-03-12

**Authors:** Şule Sanal, Sefa Yıldırım, Mehmet Yücel, Ali İlteriş Aykun, Mehmet Akif Sarı, Ayşe Menteş

**Affiliations:** 1 Department of Veterinary Medicine History and Deontology, Faculty of Veterinary Medicine, Ondokuz Mayis University, 55270 Samsun, Türkiye; suleo@omu.edu.tr; 2 Department of Veterinary Medicine History and Deontology, Institute of Graduate Studies, Ondokuz Mayis University, 55270 Samsun, Türkiye; 3 Food Technology Program, Department of Food Processing, Özalp Vocational School, Van Yüzüncü Yıl University, 65800 Van, Türkiye; mehmetyucel@yyu.edu.tr; 4 Department of Veterinary Medicine History and Deontology, Faculty of Veterinary Medicine, Erciyes University, 38039 Kayseri, Türkiye; ilterisali60@gmail.com; 5 Laboratory and Veterinary Health Program, Department of Veterinary, Alucra Turan Bulutçu Vocational School, Giresun University, 28700 Giresun, Türkiye; mehmet.akif.sari@giresun.edu.tr; 6 Department of Veterinary Medicine History and Deontology, Faculty of Veterinary Medicine, Harran University, 63200 Şanlıurfa, Türkiye; mentesgurler@gmail.com

**Keywords:** attitudes toward animals, animal hospital staff, animal attitude scale, Türkiye, veterinary services

## Abstract

This study examined general attitudes toward animals among staff working in licensed animal hospitals in Türkiye. Using the 10-item Animal Attitude Scale (AAS-10), an online survey was administered to animal hospital staff; 193 questionnaires were completed from 17 provinces. Because total scores deviated from normality, group comparisons were conducted using non-parametric tests, and a multiple linear regression model was fitted to examine joint associations with demographic and professional variables. Overall, participants reported generally positive attitudes (mean AAS-10 = 36.7 ± 5.85; range 10–50). Women scored higher than men (*p* < 0.001), and respondents aged 20–29 years scored higher than those aged ≥40 years (*p* = 0.029) in unadjusted comparisons; however, the age pattern was small and did not persist after adjustment for gender and other covariates. Professional variables, including occupational role and length of service, were not meaningfully associated with total scores. Exploratory item-level analyses suggested gender-related differences in acceptance of specific forms of animal use, but these should be interpreted cautiously given multiple comparisons. These findings provide a descriptive baseline of AAS-10 scores in a heterogeneous animal hospital workforce and support hypothesis generation for future research that incorporates practice-specific measures.

## Simple Summary

Little is known about general attitudes toward animals among staff working in licensed animal hospitals in Türkiye. We surveyed 193 veterinarians and other hospital personnel across 17 provinces using the 10-item Animal Attitude Scale (AAS-10). Overall, respondents tended to report supportive attitudes toward animal protection, although scores varied across individuals. Women scored higher than men. Age-group differences were small and should be interpreted cautiously given voluntary participation and the absence of key contextual measures. Scores did not differ meaningfully by occupational role or length of service. These findings provide a descriptive baseline and support hypothesis generation for future research using practice-specific measures.

## 1. Introduction

The legal status of animals, human responsibilities toward them, offenses committed against animals, and the penalties imposed for such offenses have been topics of concern even in the earliest legal codes [1]. With the publication of the Brambell Report in 1965 [2] and the proclamation of the Universal Declaration of Animal Rights in 1978, discussions surrounding animal welfare and animal rights gained greater depth and international visibility. In Türkiye, the signing of the European Convention for the Protection of Pet Animals in 2003 was followed by the enactment of the Animal Protection Law in 2004. This law constituted the first comprehensive national framework aimed at safeguarding all animal species and establishing fundamental principles and obligations to prevent cruelty and mistreatment. Subsequent regulatory instruments helped reinforce this framework and assigned specific responsibilities to public authorities and individuals to ensure animal welfare.

Growing awareness of the significance of animals in human life has prompted researchers to develop a range of instruments designed to assess various dimensions of our relationships with other species. According to Templer & Arikawa [3], the first published measure of the human–animal bond was the Pet Attitude Scale introduced in 1981, and since then the body of literature on human–animal interactions has expanded considerably. Within this expanding field, instruments assessing human–animal relationships have explored not only demographic variables such as gender, age, race, religious beliefs, education level, marital status, and income, but also more specific topics ranging from the effects of bird ownership on quality of life to childhood cruelty toward animals [4,5].

When the topic is animal welfare, veterinarians have long been regarded as the primary experts [6]. By the mid-1970s, members of the veterinary community had begun to recognize a shift in societal attitudes toward animals, particularly companion animals, which contributed to the introduction of the first formal ethics course in veterinary education in 1978 [7]. Veterinary ethics training, often closely associated with animal welfare [8], is now incorporated into the curricula of several veterinary faculties in Türkiye and across Europe [9,10]. According to Menor-Campos et al. [11], assessing veterinarians’ attitudes toward animals is essential for evaluating the effectiveness of animal welfare and ethics education. Numerous studies have examined animal rights, animal welfare, or attitudes toward animals specifically among veterinarians and veterinary students [11,12,13,14,15,16,17,18,19,20,21,22,23,24], and at least one study has extended this focus to include other personnel working within veterinary services [25].

Research conducted in Türkiye on the attitudes of veterinarians and veterinary students toward animals has shown that several variables influence these perspectives. İzmirli and Yaşar [22] reported that both veterinarians and veterinary students strongly supported welfare-oriented husbandry systems, and that veterinarians, in particular, demonstrated greater sensitivity to such issues than animal owners and consumers. Similarly, Ozen et al. [21] found that female veterinarians expressed more positive attitudes toward animals’ right to life than their male colleagues. Sabuncuoglu and Coban [20], evaluating Turkish veterinarians’ views within the context of European Union legislation, observed that although veterinarians generally endorsed animal welfare practices, they expressed lower levels of support for certain specific issues, such as the pre-slaughter stunning of ruminants or the discontinuation of cage-based egg production. Demographic factors, particularly education level and gender, appear to be influential in shaping these attitudes. For instance, Çavușoglu and Uzabaci [19] noted that early-year veterinary students held views that were more -oriented toward animal welfare than those in later years, suggesting that sensitivity may decline as veterinary training progresses. Taken together, these findings indicate that while the veterinary community in Türkiye demonstrates a broad concern for animals, the degree of this concern varies depending on individual characteristics and educational experiences.

Beyond the veterinary community, public concern for animal welfare in Türkiye has been examined in relation to demographic characteristics. In a large community sample, Özkul et al. [26] reported that older cohorts (particularly those over 40) showed more favourable attitudes toward animal rights than younger participants in a large Turkish community sample; notably, the authors also stated that this age-related pattern did not align with several international studies reporting greater sensitivity among younger people. These population-level findings provide a rationale for examining whether demographic characteristics (e.g., age and gender) are associated with general attitudes toward animals among staff employed in licensed animal hospitals. Animal hospitals constitute workplaces characterised by frequent human–animal contact and a diversity of professional roles operating under the same organisational context. However, the present study focuses on general attitudes as captured by the AAS-10 and does not assess practice-specific decision-making or behaviour.

Licensed animal hospitals represent heterogeneous clinical work environments in which veterinarians work together with technical and support staff. Unlike studies that focus solely on veterinarians or students, examining this setting allows for the investigation of attitudinal variability across roles within a shared organizational environment. Because these roles differ in terms of educational background, responsibilities, and patterns of contact with animals, general attitudinal differences between roles are plausible; however, the sharing of workplace norms and repeated exposure to animals may attenuate these differences. To date, no study has examined attitudes toward animals using a mixed workforce sample within the context of licensed animal hospitals in Türkiye; consequently, this professional setting remains insufficiently described empirically.

Licensed animal hospitals are shared workplaces where staff with different roles work under similar organisational routines and constraints. In this study, we focus specifically on general attitudes toward animals as captured by the AAS-10. The AAS-10 reflects broad evaluative orientations toward animals and animal use; it does not measure ethical reasoning in clinical dilemmas, moral judgment, institutional ethical climate, or actual behaviour. Accordingly, the study aims to describe how general animal attitudes vary across demographic and occupational characteristics within the participating sample.

In this study, we aimed to describe general attitudes toward animals among veterinarians and other staff working in licensed animal hospitals across Türkiye and examine whether AAS-10 total scores varied by key demographic and professional characteristics (gender, age group, professional role, and length of service). We also report the scale’s performance in this sample to inform interpretation of subgroup comparisons, given that the AAS-10 captures general attitudinal orientation rather than practice-specific clinical decision-making.

## 2. Materials and Methods

### 2.1. Data Collection Tool

The Animal Attitude Scale (AAS) was developed to assess individual differences in general attitudes toward the human use of other species and concern for animal protection [27]. A brief 10-item version (AAS-10) was derived from the original 20-item scale to provide a shorter measure that remains highly correlated with the AAS [28]. In the present study, we used the AAS-10 as a measure of general attitudinal orientation toward animal use and protection across societal contexts, rather than as a tool designed to capture clinic-specific ethical dilemmas.

Items were rated on a five-point Likert scale (1 = strongly disagree, 5 = strongly agree). Negatively keyed items (items 2, 3, 4, 7, and 8; marked with an asterisk in Table S1) were reverse-coded prior to calculating the total score. Responses were then summed to obtain a total score ranging from 10 to 50, with higher scores indicating more positive attitudes toward animals. The AAS-10 items were translated into Turkish by the research team and administered accordingly, and the entire procedure was approved by the ethics committee. Because a direct translation was used, measurement equivalence with the original instrument cannot be assumed. Therefore, the findings should not be interpreted as a formal validation of a Turkish version of the scale, but rather as descriptive estimates specific to the study sample.

### 2.2. Data and Sample Collection

The study population comprised staff employed in the 91 animal hospitals licensed by the Ministry of Agriculture and Forestry in Türkiye. Recruitment followed a two-stage approach. First, each hospital on the Ministry list was contacted by telephone, and an authorised representative (e.g., a manager or senior veterinarian) was invited to forward a standardised invitation message containing the survey link to staff members. Second, to increase reach in major metropolitan areas, a subset of hospitals in Istanbul and Ankara was visited, and the same invitation and survey link were shared again. Because recruitment was mediated by institutional gatekeepers and hospital identifiers were not collected, no hospital-level dissemination log was maintained. As a result, it is not possible to determine which hospitals forwarded the survey, which declined to do so, or how many unique hospitals were ultimately represented in the sample. Accordingly, a formal response rate could not be calculated, and the sample should be regarded as a voluntary convenience sample.

The questionnaire was prepared and administered via Google Forms, and 193 participants completed it. Information on veterinarians’ primary practice focus or species domain (e.g., predominantly companion animal versus farm animal caseload) was not collected; therefore, analyses could not be stratified by the nature of clinical practice. Respondents were based in 17 provinces: Ankara (*n* = 43, 22.3%), Samsun (*n =* 24, 12.4%), Kayseri (*n =* 22, 11.4%), Erzurum (*n =* 19, 9.8%), Muğla (*n =* 14, 7.3%), Aydın (*n =* 10, 5.2%), Istanbul (*n =* 10, 5.2%), Giresun (*n =* 9, 4.7%), Antalya (*n =* 7, 3.6%), Balıkesir (*n =* 7, 3.6%), Sivas (*n =* 6, 3.1%), Iğdır (*n =* 5, 2.6%), Kars (*n =* 5, 2.6%), Diyarbakır (*n =* 4, 2.1%), Bingöl (*n =* 4, 2.1%), Elazığ (*n =* 2, 1.0%), and Mersin (*n =* 2, 1.0%). These province-level data provide only a conservative lower bound on institutional coverage, as at least one hospital must have contributed from each province, but the true number of participating hospitals remains unknown. The survey was completed anonymously, no personally identifying information was collected, and responses were analysed in aggregate. Informed consent was obtained on the introductory page of the questionnaire, and completion of the survey was taken to indicate voluntary agreement to participate. Data collection took place between April 2023 and January 2024.

### 2.3. Ethics Approval

The study was carried out with the approval of Ondokuz Mayis University Social and Human Sciences Research Ethics Committee, with the decision dated 27 January 2023, and numbered 2023-1293.

### 2.4. Statistical Analysis

The data were analysed using IBM SPSS Statistics v23 (IBM Corp., Armonk, NY, USA) and the R software v4.5.2 (R Foundation for Statistical Computing, Vienna, Austria). Distributional assumptions were evaluated using graphical inspection and the Shapiro–Wilk test. Because total AAS-10 scores deviated from normality, group comparisons were conducted using the Mann–Whitney U test for gender and the Kruskal–Wallis H test for age group, professional role, and length of service, with Dunn–Bonferroni post hoc comparisons as needed. For non-parametric tests, effect sizes were reported as rank-biserial correlation (Mann–Whitney U) and epsilon-squared (e^2^) for Kruskal–Wallis tests. To examine joint associations of demographic and professional variables with total AAS-10 scores, we fitted an ordinary least squares regression model treating the summed score as a quasi-continuous index. We retained this approach because the 10-item total score covered 41 observed values in the present sample (10–50) and is commonly analysed as approximately continuous for mean structure comparisons; however, because residual normality was imperfect, inferential emphasis was placed on robust standard errors and sensitivity analyses rather than on strict normal-theory assumptions. In addition, we conducted standard regression diagnostics to assess model adequacy. Specifically, we inspected residual versus fitted plots and normal Q-Q plots, and we formally assessed residual normality using the Shapiro–Wilk test. Potential heteroskedasticity was evaluated using both the Breusch–Pagan and White tests. We also examined leverage and influence diagnostics, including studentized residuals and Cook’s distance, to identify potentially influential observations. These checks motivated the use of heteroskedasticity-consistent (HC3) standard errors and the sensitivity analyses reported below. Because residual normality was imperfect, we report heteroskedasticity-consistent (HC3) standard errors and 95% confidence intervals, and we conducted sensitivity analyses using cluster-robust standard errors by province and median quantile regression. Item-level response distributions were compared across age and gender using Pearson chi-square or Fisher–Freeman–Halton tests as appropriate. Given multiple item-level tests, *p*-values were adjusted using the Benjamini–Hochberg false discovery rate procedure across the 10 items separately for age and gender; adjusted *p*-values are reported in Table S2 in parentheses. Multicollinearity was assessed using variance inflation factors (VIFs). Results were reported as n (percentage) for categorical variables and as mean ± standard deviation or median (minimum–maximum) for quantitative variables. Statistical significance was set at *p* < 0.05.

In the regression model, the reference categories were male (gender), 20–29 years (age), less than 1 year (length of service), and other (professional role), selected to facilitate interpretation of the regression coefficients.

In addition to Cronbach’s alpha, we estimated ordinal reliability (ordinal alpha based on polychoric correlations) and McDonald’s omega for the AAS-10 total score. As a sensitivity analysis for ordinal item responses, we conducted exploratory factor analysis using polychoric correlations (minimum residual extraction; one-factor solution).

## 3. Results

### 3.1. Demographic Characteristics

Examination of the participants’ demographic characteristics (Table 1) showed that the number of male respondents was higher than that of female respondents. The largest age group consisted of individuals between 20 and 29 years of age, followed by those aged 30–39 years and those aged 40 years and older. With respect to professional roles, veterinarians represented the highest proportion of participants, and the most common length of service was 1–5 years. Thus, the sample predominantly comprised relatively young veterinarians and early-career staff working in animal hospitals.

### 3.2. Animal Attitude Scale

For the total sample (*n =* 193), the mean score on the Animal Attitude Scale-10 (AAS-10) was 36.7 (SD = 5.85), and the median was 37, with scores ranging from 10 to 50. Overall, participants tended to report generally positive attitudes toward animals, with substantial inter-individual variation. Item response distributions were also skewed for several items, with ceiling effects on widely endorsed statements (Table S1), which restricts variance and can attenuate inter-item correlations and reliability estimates. Internal consistency of the total score was modest (Cronbach’s α = 0.689), and corrected item-total correlations ranged from 0.22 to 0.50 (Table S1). To examine whether the modest internal consistency was driven by a single problematic item, we conducted item deletion diagnostics. Removing any single item did not increase Cronbach’s alpha. Alpha if item deleted ranged from 0.635 to 0.687, which is equal to or lower than the full-scale estimate (alpha = 0.689). This pattern suggests that the modest reliability reflects diffuse inter-item covariance rather than a single poorly performing item. Given that the AAS-10 was developed as a brief measure spanning multiple animal use contexts, some reduction in internal consistency relative to longer and more homogeneous scales is expected, and alpha should be interpreted cautiously because it is sensitive to test length and departures from strict unidimensionality. Exploratory factor-analytic indicators are presented below and suggest a weak general factor; therefore, subgroup comparisons should be interpreted cautiously and primarily as descriptive.

There was a statistically significant difference in median total AAS-10 scores across age groups (*p* = 0.029; Table 2), but the magnitude was small (e^2^ = 0.027). The median score was 39 among participants aged 20–29 years, 37 among those aged 30–39 years, and 35 among those aged ≥40 years. Post hoc Dunn–Bonferroni tests indicated that the youngest group (20–29 years) scored higher than the oldest group (≥40 years). No significant differences in total scores were observed across categories of length of service (*p* = 0.238; e^2^ = 0.006) or professional roles (*p* = 0.139; e^2^ = 0.018). A gender difference was observed (*p* < 0.001; Table 2) with women showing higher scores than men; the corresponding effect size was moderate-to-large (rank-biserial r = −0.42, indicating higher scores among women).

### 3.3. Exploratory Factor Analysis

Sampling adequacy was acceptable (KMO = 0.74), and Bartlett’s test of sphericity was significant, χ^2^(45) = 301.5, *p* < 0.001, confirming the suitability of the data for factor analysis. Exploratory factor-analytic diagnostics indicated a dominant first factor, but the evidence for strict unidimensionality was limited. The first eigenvalue (2.72) was clearly larger than the subsequent ones and the scree plot showed an elbow after the first factor (Figure S1); however, the explained variance was modest (27.1%), additional eigenvalues slightly above 1.0 were present, and the item loadings in Table S1 were generally low. Taken together, this pattern is better interpreted as indicating a weak general factor than as demonstrating a clearly one-dimensional structure in this sample.

As a sensitivity analysis for ordinal items, polychoric-based estimates were computed. Ordinal alpha (0.692) and McDonald’s ωt (0.696) were comparable to Cronbach’s alpha. A polychoric one-factor solution yielded loadings of 0.255–0.616 and accounted for 19.3% of the variance, supporting a weak general factor.

### 3.4. Multiple Linear Regression Analysis

To examine the joint associations of demographic and professional variables with total AAS-10 scores, a multiple regression model was fitted with the total score as the dependent variable (Table 3). The overall model was statistically significant (F = 3.183, *p* = 0.001), although the explained variance was modest (R^2^ = 0.162), indicating that the predictors accounted for a limited share of variability in attitudes. Using HC3 standard errors, gender remained the only variable significantly associated with total AAS-10 scores: female participants scored higher than male participants (B = 4.054, *p* < 0.001). Age group, length of service, and professional role were not significantly associated with total scores (all *p* > 0.05). Sensitivity analyses using cluster-robust standard errors by province and median quantile regression yielded the same substantive conclusion (gender remained significant; age remained non-significant).

Regression diagnostics indicated deviations from residual normality (Shapiro–Wilk W = 0.972, *p* = 0.0006). Heteroskedasticity tests were mixed; the Breusch–Pagan test was not significant (*p* = 0.607), whereas the White test suggested heteroskedasticity (*p* = 0.012), supporting the use of HC3 robust standard errors. Influence diagnostics did not indicate highly influential observations (maximum Cook’s distance = 0.140, with no values approaching 1). One respondent had an extreme low total score (AAS-10 = 10) and a large negative studentized residual (approximately −4.61), but excluding this observation did not change the substantive conclusions. Sensitivity analyses using province cluster-robust standard errors and median quantile regression likewise yielded the same substantive pattern, with gender remaining significant and age remaining non-significant. For example, in median quantile regression, the estimated female versus male difference was 3.5 points (*p* < 0.001), consistent with the OLS estimates.

### 3.5. Differences Across Age Groups

Item-level analyses suggested age-related differences for the pets item and cosmetic testing item in unadjusted tests (Table S2). However, after false discovery rate adjustment across the 10 items, no age-related item differences remained statistically significant. Accordingly, item-level age patterns are presented as exploratory signals rather than confirmatory evidence.

### 3.6. Gender Differences in Scale Items

Gender differences were observed at the item level (Table S2). In unadjusted tests, women expressed less acceptance of animal use for food, medical research, dissection, and skins/leather, and lower endorsement of the human moral dominance item. After false discovery rate adjustment across the 10 items, the most robust differences remained for items related to animal use for food and human moral dominance (both adjusted *p* < 0.001), with additional adjusted differences for dissection (adjusted *p* = 0.018) and the shelter dog breeding item (adjusted *p* = 0.013). Other item-level gender differences should be interpreted cautiously given multiplicity and measurement imprecision.

## 4. Discussion

This study provides a descriptive snapshot of attitudes toward animals among staff employed in licensed animal hospitals in Türkiye who participated in this survey. Across analyses, the most consistent differences in AAS-10 scores were observed by gender and age, with women and younger participants tending to report higher scores. In contrast, professional variables such as occupational role and length of service were not statistically associated with total AAS-10 scores in this sample. Taken together, these findings suggest that demographic gradients reported in broader community studies may also be observable within animal hospital personnel; however, given the cross-sectional design and the scope of the AAS-10 as a measure of general attitudes, the results should be interpreted as descriptive associations rather than causal effects.

It is important to interpret these results in line with what was measured. The outcome in this study is a general attitude score toward animals, not a measure of ethical decision-making, clinical welfare behaviour, or compliance with professional standards. Therefore, any observed group differences should be read as differences in broad attitudinal orientation rather than evidence about how participants would act in specific veterinary ethical dilemmas or workplace situations.

The AAS-10 does not assess attitudes toward practice-specific veterinary ethical dilemmas. Instead, it captures general attitudes toward the ethical use and treatment of animals across multiple societal domains. Accordingly, the present findings should not be interpreted as direct evidence about clinical decision-making or the quality of care provided in animal hospitals. The social psychological literature shows that attitudes alone do not determine behaviour. According to the Theory of Planned Behavior, attitudes influence behaviour only indirectly through behavioural intentions and in conjunction with contextual factors such as perceived behavioural control and subjective norms [29]. Moreover, empirical research has demonstrated that the association between intentions and actual behaviour is modest and that a systematic gap exists between these two levels [30]. In this framework, AAS-10 scores can at most be interpreted as indicators of a general ethical orientation. It cannot be assumed that this orientation translates directly or deterministically into behaviour in clinical practice. Clinical decisions are strongly shaped by institutional regulations, professional norms, and practical constraints. Recent evidence from veterinarians and veterinary students working in dairy cattle contexts likewise suggests that favourable attitudes and professional values toward animal welfare do not automatically translate into a perceived capacity to influence practice, underscoring the importance of practical constraints and professional control in shaping welfare-related action [31].

The modest internal consistency and weak general factor indicate non-trivial measurement error and limited unidimensionality in this occupational sample. This has two implications for interpretation. First, measurement error primarily reduces precision and statistical power for detecting subgroup differences, so null or small effects should not be overinterpreted as evidence of true equivalence. Second, given the weak one-factor structure, we treat the AAS-10 total score as a descriptive composite index rather than a precise latent trait estimate. We did not modify the standardized AAS-10 item set post hoc because item trimming without a dedicated validation study risks capitalising on chance, undermining content coverage, and reducing comparability with prior AAS-10 applications. Future research should conduct a formal validation of the Turkish version, including confirmatory factor analysis, tests of measurement invariance across key groups, and evaluation of item functioning.

When total scale scores were compared across groups (Table 2), differences were observed for age and gender, whereas no statistically significant differences were detected for professional role or length of service. Prior studies often, but not uniformly, report more welfare-supportive orientations among younger participants [32,33,34,35,36,37], while other work finds weak, irregular, or null associations [38,39,40]. Evidence from the Turkish public also appears mixed; Özkul et al. [26], for example, reported more positive attitudes among participants aged over 40 years. In the present dataset, the youngest age group showed higher mean scores in unadjusted comparisons (Table 2), and age-group differences were visible in unadjusted item-level tests for selected content categories (Table S2). However, age did not emerge as an independent predictor in the multivariable regression (Table 3), suggesting that any bivariate age pattern is modest and may partly reflect sample composition or residual confounding. Accordingly, age-related differences should be interpreted as descriptive signals rather than as evidence of a stable age effect in this occupational population.

Although no statistically significant differences were observed based on professional role or length of service, participants with more than five years of experience had slightly lower mean scores, but this difference was not statistically meaningful. Prior work likewise reports limited or inconsistent associations between experience and attitudes/empathy toward animals among veterinary personnel [25,41]. In the present study, professional role categories were necessarily broad and did not capture primary practice focus, caseload, or species domain; these unmeasured dimensions may be more directly related to attitudes than job title per se. This interpretation is supported by recent evidence from veterinary students in the Netherlands, in whom attitudes differed systematically across animal categories (pet, pest, and profit) and also varied according to career choice, background, and diet, suggesting that species-related orientation may be more informative than broad role labels alone [42]. Accordingly, the null associations for professional variables should not be interpreted as evidence of attitudinal equivalence across practice contexts, but as an absence of detectable differences within the constraints of the current measurement and sampling design.

Studies consistently report gender differences in attitudes toward animals, with women generally expressing more welfare-supportive orientations than men [27,43]. Consistent with this pattern, women in our study obtained higher total scale scores than men (Table 2), and gender differences were particularly evident on items representing specific content categories (Table S2). At the item level, the most robust gender differences concerned animal use for food production and general endorsement of human moral dominance, and both remained statistically significant after Benjamini–Hochberg false discovery rate correction (Table S2). Two additional items, acceptance of animal dissection for educational purposes and acceptance of breeding purebred dogs while shelter dogs are euthanized, also remained significant after false discovery rate adjustment (both adjusted *p* = 0.018). Item-level differences for medical research and skins or leather were apparent in unadjusted tests but did not survive false discovery rate correction; these should therefore be treated as exploratory signals rather than confirmatory evidence. Although these patterns are broadly consistent with prior work, we did not measure plausible explanatory mechanisms such as empathy, ethical ideology, religiosity, husbandry background, or type of animal contact; accordingly, causal or psychological interpretations should be made cautiously.

- Use of Animals in Medical Research: Female participants exhibited more critical attitudes toward animal experimentation than males. Prior literature often reports that women place greater weight on the ethical implications of animal suffering and may evaluate the necessity of such procedures more critically, whereas men may show greater acceptance when potential human benefits are emphasized [27,43,44,45]. However, our data do not allow strong inferences about the mechanisms underlying this pattern, and in our sample the item-level difference did not remain statistically significant after false discovery rate correction (Table S2). It should therefore be interpreted as exploratory.
- Use of Animals for Food Consumption: Although female participants did not reject the use of farm animals entirely, they demonstrated higher sensitivity toward welfare compared to men. In item-level analyses, women were less accepting of raising animals for human consumption, and this difference remained statistically significant after false discovery rate correction (Table S2). One possible interpretation is that attitudes toward food animal use are shaped by broader socialization patterns and by differential familiarity with utilitarian framings of animals. Previous research indicates that groups often associated with men, including farmers, hunters, and producers, frequently internalize utilitarian views that prioritize the material or economic value of animals over welfare concerns [19,46,47]. However, because we did not assess occupational background, rurality, or related variables, that explanation remains tentative in the present study.
- Human Moral Dominance: While both genders opposed unrestricted exploitation of animals, the level of opposition was markedly stronger among women. Women were also more likely to reject the claim that humans have the right to use animals as they see fit, and this item-level difference remained statistically significant after false discovery rate correction (Table S2). Our data suggest that attitudes were context-dependent. While the use of animals for food was relatively tolerated, participants across the board, and especially women, were less accepting of luxury-oriented or non-essential uses such as whaling or keeping wild animals in captivity. This pattern is consistent with the observation by Rajecki et al. [48] that tolerance for animal mistreatment varies by species and intended purpose.
- Breeding Animals for Their Skins: The use of animals for decorative or luxury purposes remains one of the least socially acceptable practices in contemporary society [49,50]. Our results were broadly in line with this pattern, although male participants appeared somewhat more accepting, or less uniformly rejecting, of the fur industry than female participants. As with medical research, however, item-level differences did not survive false discovery rate correction (Table S2) and should therefore be treated as exploratory rather than robust evidence.
- Dissection of Animals for Educational Purposes: Consistent with established research [50,51,52], our study indicates a gendered difference in attitudes toward the acceptability of animal dissection in educational settings. Male participants were generally more accepting of animal dissection as a pedagogical practice, whereas female participants were more likely to reject it. This item-level difference remained statistically significant after false discovery rate adjustment (adjusted *p* = 0.018; Table S2). Even so, our data do not identify the reasons for this difference and should not be used to infer specific motivational or ethical mechanisms.
- Attitudes Toward Pet Animals: A significant ethical dissonance was observed regarding the euthanasia of shelter dogs alongside the continued breeding of purebreds. Women expressed much stronger opposition to this practice, and the item-level difference remained statistically significant after false discovery rate adjustment (adjusted *p* = 0.018; Table S2). Such differences are sometimes interpreted in light of variation in empathic responsiveness or moral concern toward companion animals, but in the present study this remains speculative because we did not measure the relevant constructs. As argued by Mestre et al. [53] and Robbins et al. [18], women often exhibit more robust empathetic responses toward companion animals, a pattern that may strengthen with age and social maturation.

A further interpretive constraint concerns what is (and is not) captured by commonly used attitude instruments. Reviews of human–animal relationship measures note a strong emphasis on companion animals [5], and this emphasis may shape which domains are most reliably assessed. One plausible contributor is the distinctive position of companion animals within households, where they are often treated with affection and framed as family members in ways comparable to young children [54]. In addition, rising rates of pet ownership and the growing number of companion animals in recent years may further increase the everyday salience of this domain and, by extension, its prominence in research applications [55]. In contemporary urban contexts, limited direct contact with animals used for food and the routinisation of industrial production may reduce opportunities for lay attitudinal elaboration in these domains [56]. In the current study, we did not measure participants’ contact histories, practice focus, or exposure to specific animal use contexts; therefore, any account of why certain content categories appeared more salient should be treated as contextual interpretation rather than an explanation supported by the present data.

More broadly, attitudes toward animals are embedded in legal, cultural, and religious frameworks that vary across societies. This broader point is consistent with recent comparative evidence from veterinary and animal welfare professionals in the United Kingdom and Japan, showing that culturally distinct professional contexts can be associated with different understandings of animal welfare principles and different evaluations of animal management practices [57]. Türkiye has several context features that are frequently discussed in this literature, including religious/ethical discourse that condemns cruelty and emphasises humane treatment [58,59], and the high visibility of free-roaming dogs and cats in many cities, which structures everyday human–animal contact [60]. Such features could, in principle, relate to how moral concern is allocated across species and use contexts, as suggested by work on moral expansiveness and contact [61]. However, the present study did not measure religiosity, moral foundations/ethical ideologies, or the frequency and type of contact with free-roaming animals. We therefore avoid treating these contextual features as mechanisms for the observed patterns and instead present them as hypotheses for future studies that explicitly operationalise and test these constructs.

Several limitations warrant acknowledgement. First, recruitment relied on institutional gatekeepers, and we intentionally did not collect hospital identifiers or track dissemination to protect organisational confidentiality and to minimise any perception of employer monitoring or coercion. This design choice precluded estimation of the number of contributing hospitals, calculation of a conventional response rate, and assessment of differential participation across hospital types. In practice, dissemination was not uniformly permitted, particularly in some public and municipality-affiliated hospitals, which may have contributed to undercoverage, self-selection, and well-recognised nonresponse bias challenges in web-based surveys [62,63]. Accordingly, the results should be interpreted as descriptive patterns among respondents rather than as population-level estimates for all staff employed in licensed animal hospitals in Türkiye. Geographic participation was also uneven, and the cross-sectional design restricts interpretation to associations rather than causal inferences [64]. Second, responses may be correlated within provinces and hospitals. Because hospital identifiers were not collected, we could not model within-hospital clustering directly (for example, multilevel models with hospital random effects) or quantify the extent of within-hospital dependence. We therefore treated observations as independent in the primary analyses and complemented regression estimates with cluster-robust standard errors by province as a sensitivity check; however, province-level clustering is an imperfect proxy for hospital-level correlation and does not eliminate the possibility of residual dependence. Third, we did not differentiate practitioner types by primary practice focus or species domain; this unmeasured heterogeneity may have obscured role-related differences and may partly explain null findings for professional variables. Fourth, key contextual variables that plausibly shape attitudes in this setting, such as frequency and type of contact with animals and measures of local cultural background (for example, religiosity and contact with free-roaming animals), were not collected, limiting contextual explanation. Finally, measurement limitations should be emphasised. Because the AAS-10 is a general attitude measure, several items have limited direct relevance to routine animal hospital decision contexts, and the scale should not be interpreted as a measure of attitudes toward specific clinical welfare dilemmas. Although Cronbach’s alpha is often benchmarked against conventional thresholds, it has well-documented limitations as a stand-alone index of measurement quality [65]. Accordingly, we complemented alpha with ordinal reliability estimates and polychoric sensitivity analyses that are more appropriate for ordinal item response formats [66,67]. Psychometric performance was modest: internal consistency was 0.689 (Cronbach’s alpha), with comparable ordinal estimates (ordinal alpha = 0.692; omega_t = 0.696). Sensitivity analyses further suggested a weak general factor (polychoric one-factor solution: loadings 0.255 to 0.616; variance explained approximately 19.3%), indicating non-trivial measurement error and limited precision for subgroup comparisons. Accordingly, subgroup differences and regression coefficients should be interpreted as descriptive patterns within the participating sample rather than robust evidence of population-level estimates for all animal-hospital staff in Türkiye. In addition, the AAS-10 was administered using a Turkish translation prepared for this study, and no formal cross-cultural adaptation or linguistic validation was conducted. Therefore, strict measurement equivalence with the original version cannot be assumed, and observed subgroup differences may partly reflect translation-related or contextual interpretation differences. Future work should implement established cross-cultural adaptation procedures and validate the Turkish version using confirmatory factor analysis and measurement invariance testing. From a sampling perspective, future studies would also benefit from stratified recruitment and maintaining a minimal institutional participation log (recording contact and willingness to disseminate without linking institutions to individual responses) to enable clearer coverage assessment.

## 5. Conclusions

This study provides a descriptive profile of AAS-10 attitudes toward animals among a heterogeneous sample of staff employed in licensed animal hospitals in Türkiye. Across analyses, gender showed the most consistent association with the total score. Age differences were detectable in unadjusted group comparisons, but the effect was small and did not persist after adjustment for gender and other covariates. Professional role and length of service were not associated with total scores; however, this null pattern should be interpreted cautiously because primary practice focus/species domain was not measured and recruitment was geographically uneven.

The regression model explained a modest proportion of variance in AAS-10 scores (R^2^ approximately 0.16), and the study design and measurement characteristics support an interpretation of the findings as descriptive, sample-specific associations rather than causal effects or population-level estimates. Importantly, because the AAS-10 is a general attitude measure, these results should not be interpreted as direct evidence about views on specific practice-based welfare conflicts encountered in daily clinical work.

Overall, the findings provide a baseline for hypothesis generation in this understudied professional context. Future studies should employ more structured sampling, measure practice context (e.g., caseload/species domain, animal contact frequency), and incorporate validated Turkish instruments or formally adapted versions to improve measurement equivalence. Where feasible, designs that account for clustering within hospitals and prospective follow-up would strengthen inference about how general ethical orientations relate to professional outcomes (e.g., compassion fatigue, well-being) and clinical decision-making.

## Figures and Tables

**Table 1:** Descriptive Statistics of Demographic Variables.

**Gender**	***n***	***n%***
Male	109	56.5
Female	84	43.5
**Age**	***n***	***n%***
20–29	87	45.1
30–39	59	30.6
40 and over	47	24.4
**Professional roles**	***n***	***n%***
Veterinarian	59	30.6
Veterinary specialist	47	24.4
Responsible manager (veterinarian)	25	13
Veterinary technician	22	11.4
Medical laboratory technician	19	9.8
Other	21	10.9
**Length of service**	***n***	***n%***
Less than 1 year	33	17.1
1–5 years	88	45.6
5–10 years	33	17.1
More than 10 years	39	20.2

**Table 2:** Comparison of Total Animal Attitude Scale Scores by Groups.

	Scale Total Score		Test Statistic	*p*	Effect Size
	Mean ± SD	Median (Min–Max)			
**Gender**					r_rb = −0.42
Male	34.81 ± 5.73	36 (10–44)	2653.5 *	<0.001	
Female	39.17 ± 5.07	39 (27–50)			
**Age**					e^2^ = 0.027
20–29	37.56 ± 5.95	39 (10–50) ^a^	7.075 **	0.029	
30–39	36.78 ± 5.5	37 (25–47) ^a,b^			
40 and over	35.02 ± 5.87	35 (23–50) ^b^			
**Professional roles**					e^2^ = 0.018
Veterinarian	37.88 ± 5.4	39 (23–50)	8.333 **	0.139	
Veterinary specialist	35.36 ± 5.3	35 (23–46)			
Responsible manager (veterinarian)	35.08 ± 7.85	36 (10–50)			
Veterinary technician	37.68 ± 4.95	37 (28–47)			
Medical laboratory technician	36.74 ± 6.18	39 (24–46)			
Other	37.29 ± 5.71	38 (27–44)			
**Length of service**					e^2^ = 0.006
Less than 1 year	37.21 ± 5.94	37 (27–47)	4.223 **	0.238	
1–5 years	37.38 ± 5.97	38 (10–50)			
5–10 years	35.82 ± 5.37	36 (25–47)			
More than 10 years	35.51 ± 5.83	36 (23–44)			

* Mann–Whitney U test (gender); ** Kruskal–Wallis H test (age, professional roles, and length of service). Dunn–Bonferroni post hoc tests were used; groups sharing the same superscript letter do not differ significantly. Effect sizes: rank-biserial correlation (r_rb) for Mann–Whitney U; epsilon-squared (e^2^) for Kruskal–Wallis.

**Table 3:** Multiple Linear Regression Analysis of Factors Associated with Total AAS-10 Scores.

	B (95% CI)	SE	β	t	*p*	VIF
**Constant**	35.507 (31.403–39.611)	2.080	-	17.072	<0.001	
**Gender (Reference: Male** ) *						
Female	4.054 (2.307–5.800)	0.885	0.344	4.579	<0.001	1.139
**Age (Reference: 20–29** ) *						
30–39	0.767 (−2.118–3.652)	1.462	0.061	0.524	0.601	2.040
40 and over	−0.857 (−4.510–2.796)	1.851	−0.063	−0.463	0.644	2.663
**Length of service (Reference: less than 1 year** ) *						
1–5 years	0.419 (−1.936–2.774)	1.193	0.036	0.351	0.726	2.109
5–10 years	0.047 (−3.169–3.262)	1.630	0.003	0.029	0.977	2.368
More than 10 years	0.913 (−2.389–4.216)	1.674	0.063	0.546	0.586	2.983
**Professional roles (Reference: Other** ) *						
Veterinarian	−0.167 (−3.641–3.307)	1.761	−0.013	−0.095	0.924	3.058
Veterinary specialist	−2.195 (−5.541–1.152)	1.696	−0.161	−1.294	0.197	2.939
Responsible manager (veterinarian)	−1.431 (−5.808–2.946)	2.218	−0.082	−0.645	0.520	2.039
Veterinary technician	−0.374 (−4.291–3.543)	1.985	−0.020	−0.188	0.851	1.975
Medical laboratory technician	−1.647 (−5.495–2.202)	1.951	−0.084	−0.844	0.400	1.775

F = 3.183, *p* = 0.001; R^2^ = 0.162. B = unstandardized regression coefficient; β = standardized regression coefficient; SE = HC3 robust standard error; CI = confidence interval. * Reference categories: male (gender), 20–29 (age), less than 1 year (length of service), and other (professional role).

## Data Availability

Data sets generated during the current study are available from the corresponding author on reasonable request.

## Supplementary Materials

The following supporting information can be downloaded at: https://www.mdpi.com/article/10.3390/ani16060888/s1, Table S1: Item-level psychometric properties of the AAS-10 in the current sample; Table S2: Age- and gender-stratified differences in scale-item responses; Figure S1: Scree plot of eigenvalues for the AAS-10.

## References

[1] Menteş Gürler A., Osmanağaoğlu Ş. (2009). Türkiye’de hayvanları koruma kanununun tarihsel gelişimi. Kafkas Univ. Vet. Fak. Derg..

[2] Brambell F.W.R. (1965). Report of the Technical Committee to Enquire into the Welfare of Animals Kept Under Intensive Livestock Husbandry Systems.

[3] Templer D.I., Arikawa H., Blazina C., Boyraz G., Shen-Miller D. (2011). The pet attitude scale. The Psychology of the Human-Animal Bond: A Resource for Clinicians and Researchers.

[4] Taylor N., Signal T. (2006). Attitudes to animals: Demographics within a community sample. Soc. Anim..

[5] Wilson C.C., Netting F.E. (2012). The status of instrument development in the human-animal interaction field. Anthrozoös.

[6] Beaver B.V. (2005). Introduction: Animal welfare education, a critical time in veterinary medicine. J. Vet. Med. Educ..

[7] Rollin B., Sanal Ş., Melikoğlu Gölcü B., Doğan Ö. (2014). An Introduction to Veterinary Medical Ethics. IV. Ulusal Veteriner Hekimliği Tarihi ve Mesleki Etik Sempozyumu Bildiriler Kitabı.

[8] Magalhães-Sant’Ana M., Lassen J., Millar K.M., Sandøe P., Olsson I.A.S. (2014). Examining why ethics is taught to veterinary students: A qualitative study of veterinary educators’ perspectives. J. Vet. Med. Educ..

[9] Magalhães-Sant’Ana M., Olsson I., Sandøe P., Millar K., Casabona C.M.R., Epifanio L.E.S., Emaldi Cirión A.E. (2010). How ethics is taught by European veterinary faculties: A review of published literature and web resources. Global Food Security: Ethical and Legal Challenges.

[10] Melikoğlu Gölcü B., Ünsal Adaca A., Yerlikaya N., Özen D., Başağaç Gül R.T. (2024). A Mixed-Methods Survey of Veterinary Ethics Teaching in Turkey. J. Vet. Med. Educ..

[11] Menor-Campos D.J., Knight S., Sánchez-Muñoz C., López-Rodríguez R. (2019). Human-directed empathy and attitudes toward animal use: A survey of Spanish veterinary students. Anthrozoös.

[12] Hazel S.J., Signal T.D., Taylor N. (2011). Can teaching veterinary and animal-science students about animal welfare affect their attitude toward animals and human-related empathy?. J. Vet. Med. Educ..

[13] Heleski C.R., Mertig A.G., Zanella A.J. (2005). Results of a national survey of US veterinary college faculty regarding attitudes toward farm animal welfare. J. Am. Vet. Med. Assoc..

[14] Levine E.D., Mills D.S., Houpt K.A. (2005). Attitudes of veterinary students at one US college toward factors relating to farm animal welfare. J. Vet. Med. Educ..

[15] Calderón-Amor J., Luna-Fernández D., Tadich T. (2017). Study of the levels of human-human and human-animal empathy in veterinary medical students from Chile. J. Vet. Med. Educ..

[16] Hagelin J., Hau J., Carlsson H.-E. (2000). Attitude of Swedish veterinary and medical students to animal experimentation. Vet. Rec..

[17] Özen A., Özen R., Yaşar A., Armutak A., Bayrak S., Gezman A., Şeker İ. (2009). Attitudes of Turkish veterinary students and educators towards the moral status of animals and species rating. Kafkas Univ. Vet. Fak. Derg..

[18] Robbins J., Danielson J., Johnson A., Parsons R., Jorgensen M., Millman S. (2021). Attitudes towards animals and belief in animal mind among first-year veterinary students before and after an introductory animal welfare course. Anim. Welf..

[19] Çavuşoğlu E., Uzabacı E. (2021). Attitudes of Turkish veterinary students towards farm animal welfare. Anim. Welf..

[20] Sabuncuoğlu N., Çoban O. (2008). Attitudes of Turkish veterinarians towards animal welfare. Anim. Welf..

[21] Özen A., Öztürk R., Yaşar A., Armutak A., Başağaç T., Özgür A., Şeker İ., Yerlikaya H. (2004). An attitude of veterinary practitioners towards animal rights in Turkey. Vet. Med..

[22] İzmirli S., Yaşar A. (2010). A Survey on Animal Welfare Attitudes of Veterinary Surgeries, Veterinary Students, Animal Owners and Society in Turkey. Kafkas Univ. Vet. Fak. Derg..

[23] İzmirli S., Yiğit A., Phillips C.J.C. (2014). Attitudes of Australian and Turkish Students of Veterinary Medicine toward Nonhuman Animals and Their Careers. Soc. Anim..

[24] İzmirli S., Phillips C.J.C. (2012). Attitudes of Australian and Turkish Veterinary Faculty toward Animal Welfare. J. Vet. Med. Educ..

[25] Hellyer P.W., Frederick C., Lacy M., Salman M., Wagner A.E. (1999). Attitudes of veterinary medical students, house officers, clinical faculty, and staff toward pain management in animals. J. Am. Vet. Med. Assoc..

[26] Özkul T., Sarıbaş T., Uzabacı E., Yüksel E. (2013). A Survey to Identify the Turkish People’s Approach on Animal Rights Concept: I. Attitude Analysis by Demographic Traits. Kafkas Univ. Vet. Fak. Derg..

[27] Herzog H.A., Betchart N.S., Pittman R.B. (1991). Gender, sex role orientation, and attitudes toward animals. Anthrozoös.

[28] Herzog H., Grayson S., McCord D. (2015). Brief measures of the animal attitude scale. Anthrozoös.

[29] Ajzen I. (1991). The theory of planned behavior. Organ. Behav. Hum. Decis. Process..

[30] Sheeran P. (2002). Intention-behavior relations: A conceptual and empirical review. Eur. Rev. Soc. Psychol..

[31] Brunt M.W., Haley D.B., LeBlanc S.J., Kelton D.F. (2024). Attitudes and professional values of veterinarians and veterinary students toward positive welfare states for dairy cattle. J. Dairy Sci..

[32] Pifer L.K. (1996). Exploring the gender gap in young adults’ attitudes about animal research. Soc. Anim..

[33] Bjerke T., Ødegårdstuen T.S., Kaltenborn B.P. (1998). Attitudes toward animals among Norwegian adolescents. Anthrozoös.

[34] Randler C., Binngießer J., Vollmer C. (2019). Composite respect for animals scale: Full and brief versions. Soc. Anim..

[35] Carnovale F., Xiao J., Shi B., Arney D., Descovich K., Phillips C.J. (2022). Gender and age effects on public attitudes to, and knowledge of, animal welfare in China. Animals.

[36] Binngießer J., Wilhelm C., Randler C. (2013). Attitudes toward animals among German children and adolescents. Anthrozoös.

[37] Su B., Martens P. (2017). Public attitudes toward animals and the influential factors in contemporary China. Anim. Welf..

[38] Azahar F.A.M., Fakri N.M.R.M., Pa M.N.M. (2014). Associations Between Gender, Year of Study and Empathy Level with Attitudes Towards Animal Welfare Among Undergraduate Doctor of Veterinary Medicine Students in Universiti Putra Malaysia. Educ. Med. J..

[39] Kılıç İ., Bozkurt Z. (2013). The relationship between farmers’ perceptions and animal welfare standards in sheep farms. Asian-Australas. J. Anim. Sci..

[40] Van Poucke E., Vanhonacker F., Nijs G., Braeckman J., Verbeke W., Tuyttens F., Kaiser M., Lien M.E. (2006). Defining the concept of animal welfare: Integrating the opinion of citizens and other stakeholders. Ethics and the Politics of Food.

[41] Colombo E.S., Crippa F., Calderari T., Prato-Previde E. (2017). Empathy toward animals and people: The role of gender and length of service in a sample of Italian veterinarians. J. Vet. Behav..

[42] Dijkstra Klaasse A.V., Janssens M.R.E., Salvatori D.C.F. (2025). Pet, Pest, Profit: Patient! How Attitudes Toward Animals Among Veterinary Students in the Netherlands Differ According to Animal Categories and Student-Related Variables. Animals.

[43] Herzog H.A. (2007). Gender differences in human-animal interactions: A review. Anthrozoös.

[44] Eldridge J.J., Gluck J.P. (1996). Gender differences in attitudes toward animal research. Ethics Behav..

[45] Pifer R., Shimizu K., Pifer L. (1994). Public attitudes toward animal research: Some international comparisons. Soc. Anim..

[46] Zalaf A. (2013). Understanding Animal Welfare in the UK and Cyprus: An Investigation of Individual Differences Underlying the Behavior and Its Relation to Humane Education in Children. Ph.D. Thesis.

[47] Kellert S.R. (1980). American Attitudes Toward and Knowledge of Animals: An Update. Int. J. Stud. Anim. Probl..

[48] Rajecki D., Rasmussen J.L., Craft H.D. (1993). Labels and the treatment of animals: Archival and experimental cases. Soc. Anim..

[49] Bradley A., Mennie N., Bibby P.A., Cassaday H.J. (2020). Some animals are more equal than others: Validation of a new scale to measure how attitudes to animals depend on species and human purpose of use. PLoS ONE.

[50] Driscoll J.W. (1992). Attitudes toward animal use. Anthrozoös.

[51] Gallop G.G., Beckstead J.W. (1988). Attitudes toward animal research. Am. Psychol..

[52] Sieber J.E. (1986). Students’ and scientists’ attitudes on animal research. Am. Biol. Teach..

[53] Mestre M.V., Samper P., Frías M.D., Tur A.M. (2009). Are women more empathetic than men? A longitudinal study in adolescence. Span. J. Psychol..

[54] Shir-Vertesh D. (2012). “Flexible personhood”: Loving animals as family members in Israel. Am. Anthropol..

[55] Busch G., Schütz A., Hölker S., Spiller A. (2022). Is pet ownership associated with values and attitudes towards animals?. Anim. Welf..

[56] Steinfeld H., de Haan C., Blackburn H. (1996). Livestock—Environment Interactions: Issues and Options.

[57] Otani Y., Kanamori M., Kato H., Dwyer C.M. (2025). Cross-cultural variation in understanding of animal welfare principles and animal management practices among veterinary and animal welfare professionals in the UK and Japan. Anim. Welf..

[58] Hackett C., Stonawski M., Tong Y., Kramer S., Shi A.F., Zanetti N. (2025). Religious Composition by Country, 2010–2020.

[59] Rahman S.A. (2017). Religion and animal welfare: An islamic perspective. Animals.

[60] Hart K. (2019). Istanbul’s intangible cultural heritage as embodied by street animals. Hist. Anthropol..

[61] Amiot C.E., Bastian B. (2015). Toward a psychology of human-animal relations. Psychol. Bull..

[62] Bethlehem J. (2010). Selection Bias in Web Surveys. Int. Stat. Rev..

[63] Groves R.M. (2006). Nonresponse Rates and Nonresponse Bias in Household Surveys. Public Opin. Q..

[64] Levin K.A. (2006). Study Design III: Cross-sectional Studies. Evid.-Based Dent..

[65] Sijtsma K. (2009). On the Use, the Misuse, and the Very Limited Usefulness of Cronbach’s Alpha. Psychometrika.

[66] Gadermann A.M., Guhn M., Zumbo B.D. (2012). Estimating Ordinal Reliability for Likert-Type and Ordinal Item Response Data: A Conceptual, Empirical, and Practical Guide. Pract. Assess. Res. Eval..

[67] Zumbo B.D., Gadermann A.M., Zeisser C. (2007). Ordinal Versions of Coefficients Alpha and Theta for Likert Rating Scales. J. Mod. Appl. Stat. Methods.

