# Supplementary material for: Measuring the Attitudes of Animal Hospital Staff Toward Animals in Türkiye
Source: Animals (Basel). 2026 Mar 12;16(6):888. doi: 10.3390/ani16060888 (PMC13023321; doi:10.3390/ani16060888)
Supplement: Supplementary file 1 [file animals-16-00888-s001.zip › Table S1.pdf]

Table S1. Item-level psychometric properties of the AAS-10 in the current sample

Item (content category)	Mean	SD	Item–total r	Factor loading (1-factor)
Hunting (sport)	4.66	0.89	.28	.29
Medical research*	2.70	1.40	.33	.29
Food production*	1.92	1.10	.39	.31
Human moral dominance*	3.78	1.28	.42	.35
Slaughter of whales and dolphins	4.64	0.74	.31	.31
Zoos	4.45	1.00	.40	.35
Breeding for skins*	3.77	1.37	.34	.32
Dissection for education*	2.83	1.07	.22	.20
Shelter dogs / purebred breeding	4.00	1.15	.31	.29
Cosmetic safety testing	3.96	1.24	.50	.41

Starred items (*) are reverse scored. For starred items, means and standard deviations are shown after reverse scoring so that higher values consistently indicate more pro-animal attitudes. Factor loadings are from the one-factor exploratory solution reported descriptively in the manuscript.
