# Supplementary material for: Measuring the Attitudes of Animal Hospital Staff Toward Animals in Türkiye
Source: Animals (Basel). 2026 Mar 12;16(6):888. doi: 10.3390/ani16060888 (PMC13023321; doi:10.3390/ani16060888)
Supplement: Supplementary file 1 [file animals-16-00888-s001.zip › Table S2.pdf]

Table S2. Age- and gender-stratified differences in scale-item responses

	Age						Gender				
	20-29 n (n%)	30-39 n (n%)	40 and over n (n%)	Test statistic (χ^2/FFH)	p (raw; adj)	Cramér's V	Male n (n%)	Female n (n%)	Test statistic (χ^2/FFH)	p (raw; adj)	Cramér's V
**1. It is morally wrong to hunt wild animals just for sport.**											
Strongly Disagree	3 (3.4)	0 (0)	3 (6.4)				5 (4.6)	1 (1.2)			
Disagree	1 (1.1)	1 (1.7)	3 (6.4)				4 (3.7)	1 (1.2)			
Undecided	2 (2.3)	0 (0)	2 (4.3)	9.659 (χ^2)	0.227 (0.568)	0.224	3 (2.8)	1 (1.2)	3.685 (FFH)	0.461 (0.512)	0.138
Agree	8 (9.2)	7 (11.9)	3 (6.4)				11 (10.1)	7 (8.3)			
Strongly Agree	73 (83.9)	51 (86.4)	36 (76.6)				86 (78.9)	74 (88.1)			
**2. I do not think that there is anything wrong with using animals in medical research.**											
Strongly Disagree	14 (16.1)	9 (15.3)	6 (12.8)				12 (11)	17 (20.2)			
Disagree	15 (17.2)	9 (15.3)	7 (14.9)				15 (13.8)	16 (19)			
Undecided	19 (21.8)	8 (13.6)	10 (21.3)	4.279 (FFH)	0.831 (0.994)	0.149	18 (16.5)	19 (22.6)	9.7 (χ^2)	0.046 (0.076)	0.224
Agree	21 (24.1)	13 (22)	12 (25.5)				28 (25.7)	18 (21.4)			
Strongly Agree	18 (20.7)	20 (33.9)	12 (25.5)				36 (33)	14 (16.7)			
**3. I think it is perfectly acceptable for cattle and hogs to be raised for human consumption.**											
Strongly Disagree	4 (4.6)	1 (1.7)	2 (4.3)				3 (2.8)	4 (4.8)			
Disagree	5 (5.7)	3 (5.1)	3 (6.4)				3 (2.8)	8 (9.5)			
Undecided	17 (19.5)	8 (13.6)	8 (17)	4.357 (χ^2)	0.841 (0.994)	0.150	10 (9.2)	23 (27.4)	24.096 (FFH)	<0.001 (<0.001)	0.353
Agree	25 (28.7)	13 (22)	12 (25.5)				26 (23.9)	24 (28.6)			
Strongly Agree	36 (41.4)	34 (57.6)	22 (46.8)				67 (61.5)	25 (29.8)			
**4. Basically, humans have the right to use animals as we see fit.**											
Strongly Disagree	37 (42.5)	22 (37.3)	12 (25.5)				26 (23.9)	45 (53.6)			
Disagree	24 (27.6)	21 (35.6)	18 (38.3)				38 (34.9)	25 (29.8)			
Undecided	11 (12.6)	5 (8.5)	5 (10.6)	6.096 (χ^2)	0.646 (0.994)	0.178	13 (11.9)	8 (9.5)	24.031 (χ^2)	<0.001 (<0.001)	0.353
Agree	9 (10.3)	7 (11.9)	6 (12.8)				18 (16.5)	4 (4.8)			

Strongly Agree	6 (6.9)	4 (6.8)	6 (12.8)				14 (12.8)	2 (2.4)			
**5. The slaughter of whales and dolphins should be immediately stopped even if it means some people will be put out of work.**											
Strongly Disagree	1 (1.1)	0 (0)	2 (4.3)				2 (1.8)	1 (1.2)			
Disagree	1 (1.1)	0 (0)	0 (0)				1 (0.9)	0 (0)			
Undecided	5 (5.7)	0 (0)	4 (8.5)	9.991 (χ^2)	0.174 (0.568)	0.228	5 (4.6)	4 (4.8)	2.423 (FFH)	0.724 (0.724)	0.112
Agree	18 (20.7)	10 (16.9)	9 (19.1)				24 (22)	13 (15.5)			
Strongly Agree	62 (71.3)	49 (83.1)	32 (68.1)				77 (70.6)	66 (78.6)			
**6. I sometimes get upset when I see wild animals in cages at zoos.**											
Strongly Disagree	3 (3.4)	1 (1.7)	0 (0)				3 (2.8)	1 (1.2)			
Disagree	5 (5.7)	2 (3.4)	5 (10.6)				7 (6.4)	5 (6)			
Undecided	5 (5.7)	5 (8.5)	2 (4.3)	6.787 (χ^2)	0.554 (0.994)	0.188	10 (9.2)	2 (2.4)	8.894 (FFH)	0.053 (0.076)	0.215
Agree	12 (13.8)	8 (13.6)	11 (23.4)				22 (20.2)	9 (10.7)			
Strongly Agree	62 (71.3)	43 (72.9)	29 (61.7)				67 (61.5)	67 (79.8)			
**7. Breeding animals for their skins is a legitimate use of animals.**											
Strongly Disagree	39 (44.8)	26 (44.1)	18 (38.3)				40 (36.7)	43 (51.2)			
Disagree	21 (24.1)	10 (16.9)	12 (25.5)				26 (23.9)	17 (20.2)			
Undecided	11 (12.6)	8 (13.6)	7 (14.9)	3.067 (χ^2)	0.942 (0.994)	0.126	19 (17.4)	7 (8.3)	11.039 (χ^2)	0.026 (0.052)	0.239
Agree	8 (9.2)	7 (11.9)	6 (12.8)				16 (14.7)	5 (6)			
Strongly Agree	8 (9.2)	8 (13.6)	4 (8.5)				8 (7.3)	12 (14.3)			
**8. Some aspects of biology can only be learned through dissecting preserved animals such as cats.**											
Strongly Disagree	6 (6.9)	5 (8.5)	3 (6.4)				5 (4.6)	9 (10.7)			
Disagree	16 (18.4)	8 (13.6)	9 (19.1)				12 (11)	21 (25)			
Undecided	34 (39.1)	24 (40.7)	16 (34)	1.757 (χ^2)	0.994 (0.994)	0.095	41 (37.6)	33 (39.3)	14.004 (χ^2)	0.007 (0.018)	0.269
Agree	21 (24.1)	15 (25.4)	14 (29.8)				35 (32.1)	15 (17.9)			
Strongly Agree	10 (11.5)	7 (11.9)	5 (10.6)				16 (14.7)	6 (7.1)			
**9. It is unethical to breed purebred dogs for pets when millions of dogs are killed in animal shelters each year.**											
Strongly Disagree	2 (2.3)	0 (0)	1 (2.1)	19.073 (χ^2)	0.008 (0.075)	0.314	2 (1.8)	1 (1.2)	14.255 (FFH)	0.004 (0.013)	0.272
Disagree	10 (11.5)	5 (8.5)	14 (29.8)				20 (18.3)	9 (10.7)			

Undecided	8 (9.2)	10 (16.9)	4 (8.5)				10 (9.2)	12 (14.3)			
Agree	17 (19.5)	20 (33.9)	13 (27.7)				37 (33.9)	13 (15.5)			
Strongly Agree	50 (57.5)	24 (40.7)	15 (31.9)				40 (36.7)	49 (58.3)			
**10. The use of animals such as rabbits for testing the safety of cosmetics and household products is unnecessary and should be stopped.**											
Strongly Disagree	3 (3.4)	1 (1.7)	3 (6.4)				6 (5.5)	1 (1.2)			
Disagree	5 (5.7)	10 (16.9)	14 (29.8)				20 (18.3)	9 (10.7)			
Undecided	12 (13.8)	8 (13.6)	2 (4.3)	17.701 (χ²)	0.015 (0.075)	0.303	13 (11.9)	9 (10.7)	5.516 (FFH)	0.235 (0.294)	0.169
Agree	20 (23)	13 (22)	9 (19.1)				23 (21.1)	19 (22.6)			
Strongly Agree	47 (54)	27 (45.8)	19 (40.4)				47 (43.1)	46 (54.8)			

Test statistics are reported as Pearson χ² or Fisher–Freeman–Halton (FFH), as indicated in the test statistic column. P-values are unadjusted; Benjamini–Hochberg false discovery rate adjusted p-values across the 10 items are shown in parentheses, computed separately for age and gender.
