# Supplementary figures and images for: Measuring the Attitudes of Animal Hospital Staff Toward Animals in Türkiye
Source: Animals (Basel). 2026 Mar 12;16(6):888. doi: 10.3390/ani16060888 (PMC13023321; doi:10.3390/ani16060888)

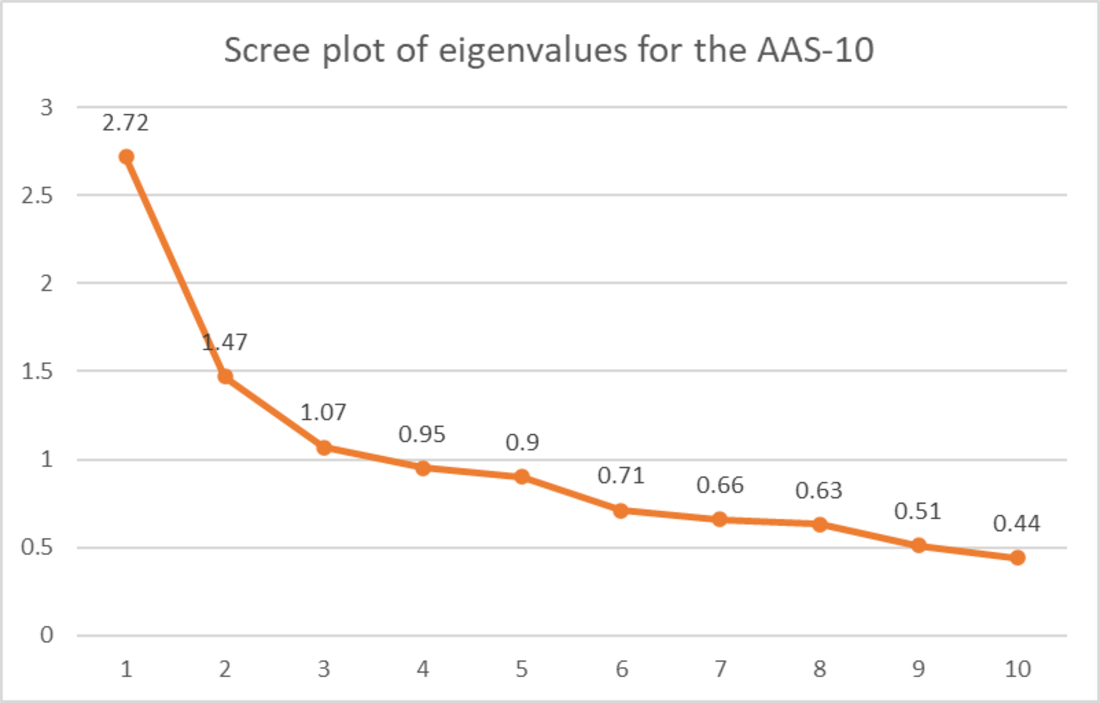

Supplement: Supplementary file 1 [file animals-16-00888-s001.zip › FIGURE S1.png]
